# Phased blood-brain barrier disruption in ischaemic stroke: implications for therapy?

**DOI:** 10.1186/s12987-025-00701-5

**Published:** 2025-08-27

**Authors:** Alissia Blase, Costanza Giovene di Girasole, Laura Benjamin, Patric Turowski

**Affiliations:** 1 UCL Institute of Ophthalmology, 11-43 Bath Street, London, EC1V 9EL UK; 2 UCL Queen Square Institute of Neurology, Queen Square, London, WCIE 3BG UK; 3 National Hospital for Neurology and Neurosurgery, Queen Square, London, WCIE 3BG UK

**Keywords:** Ischaemic stroke, Blood-brain barrier, Endothelial cell, Caveolae, Tight junctions, Reperfusion

## Abstract

Cerebrovascular disease, which primarily affects the brain’s blood vessels, remains a major global cause of death and disability. Among its clinical manifestations, ischaemic stroke is by far the most common. Prolonged oedema due to blood vessel leakage is detrimental to the delicate neuronal environment throughout the ischaemic and reperfusion phase and contributes to the mortality, morbidity, and disabilities associated with this devastating condition. Under physiological conditions, an intact blood-brain barrier (BBB) protects and regulates solute and cell transit in and out of the central nervous system. Indeed, dysfunction of this formidable cerebrovascular regulator has been functionally linked to adverse outcomes in stroke. While our knowledge of the underlying mechanism is incomplete, increasing evidence, particularly from studies using models of rodents exposed to middle cerebral artery occlusion (MCAO), supports a biphasic breakdown of the BBB in ischemic stroke. However, debate persists regarding the precise mechanisms of BBB dysfunction. Understanding this pathobiology is essential for developing targeted interventions to improve clinical outcomes in stroke patients. In this review, we provide a summary of the structure and function of the BBB as well as the cellular and molecular determinants of leakage pathways present in pathological conditions, and evaluate medical strategies aimed at reducing BBB disruption in stroke. We also discuss the potential for selectively targeting specific phases of BBB leakage.

## Introduction

Disruption of the blood-brain barrier (BBB) accompanies the sequelae of many cerebrovascular diseases, including ischaemic and haemorrhagic stroke, and cerebral amyloid angiopathy [1–3]. In the context of stroke, BBB dysfunction is not only a consequence of vascular injury but also a contributor to secondary brain damage. Increasing evidence suggests that, as predicted, an impaired BBB is functionally linked to increased vasogenic oedema and haemorrhagic transformation of an ischaemic stroke, henceforth contributing significantly to neuronal death, neurological deterioration and mortality [1]. These processes underscore the importance of understanding BBB breakdown in the acute and subacute phases of stroke to identify therapeutic windows and mitigate long-term damage.

**Fig. 1:**
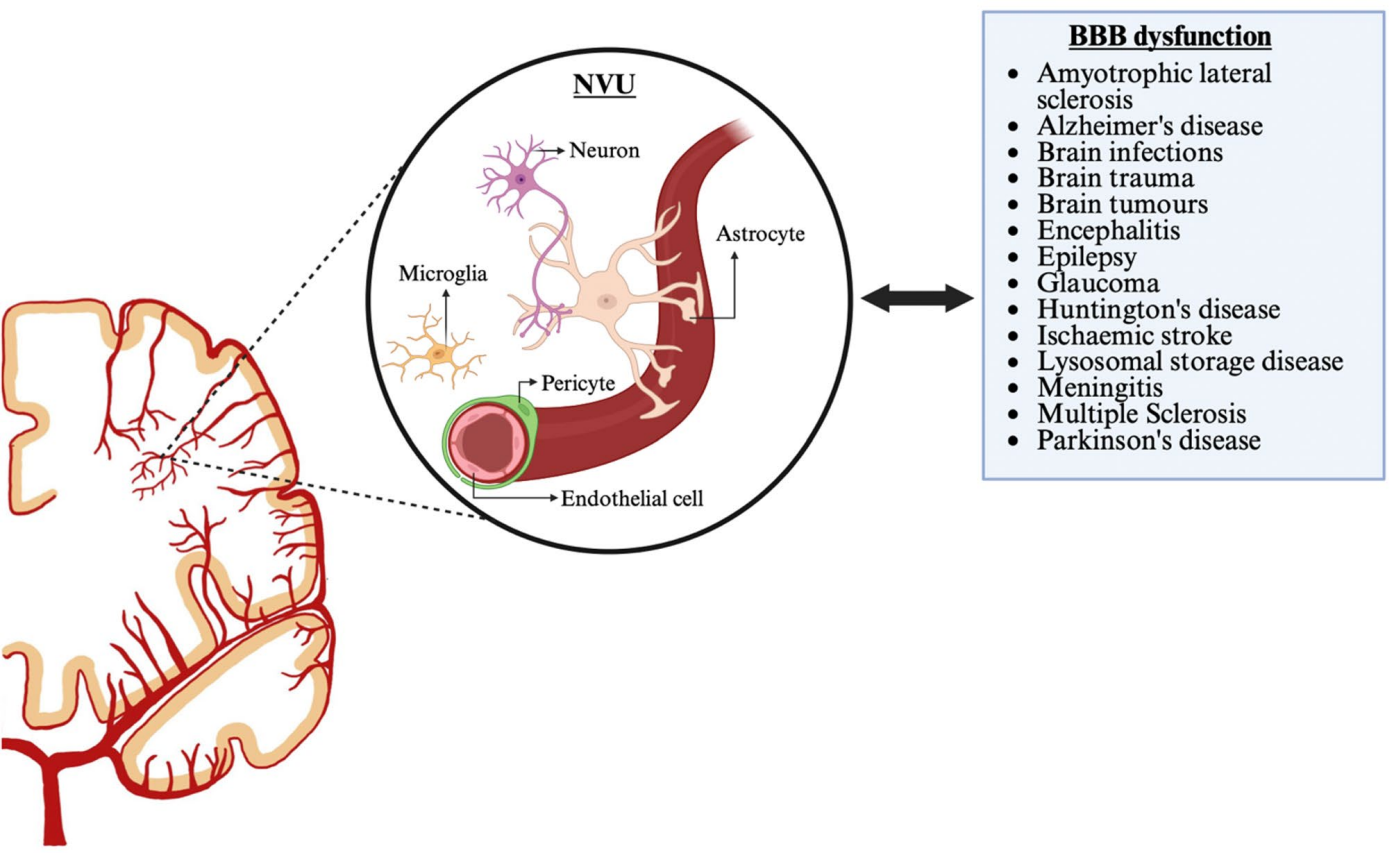
Acute and chronic neuro-degenerative and -inflammatory diseases are associated with BBB dysfunction. The neurovascular unit (NVU) defines biological activities and functional interactions of the BBB. A non-exhaustive list of diseases which are associated with a dysfunctional BBB is shown on the right. Progressive weakening of the BBB has also been observed during healthy ageing; however, this is notably accelerated in a wide variety of neurological diseases. Made with BioRender

While BBB dysfunction is more prominently observed in acute cerebrovascular events such as stroke, it is also increasingly recognised in chronic neurodegenerative and neuroinflammatory diseases, including Alzheimer’s Disease (AD), Parkinson’s disease (PD) and Multiple Sclerosis (MS) [4–7] (Fig. 1). It has also been shown to be part of ageing [8, 9]. Functional alterations of the BBB precede hallmark activity in AD and PD, with increased permeability linked to the accumulation of amyloid-β, tau and neuroinflammation [4]. These conditions provide broader insights into the progressive nature of BBB dysfunction; however, the dynamics and consequences of BBB failure in stroke are uniquely acute and mechanistically distinct. Stroke-induced BBB breakdown unfolds within hours of ischaemic onset, driven primarily by metabolic stress, oxidative injury, and neuroinflammatory signalling [10–12].

In stroke, vascular endothelial cell (EC) dysfunction at the compromised BBB involves paracellular leakage, as evidenced by the degradation of tight junction (TJ) proteins including claudin-5 (CLDN5), occludin (OCLN), and zonula occludens-1 (ZO-1/TJP1) [13,14]. Furthermore, a pivotal role for transcellular barrier dysfunction has also been described, in which increased caveolae–mediated vesicular transport facilitate the early passage of proteins and likely immune cells across the BBB [13, 15, 16]. This multifaceted breakdown of the BBB facilitates infiltration of blood solutes, circulating leukocytes, activation of CNS-resident microglia, and the accumulation of proinflammatory cytokines [10]. Leakage and immune trafficking exacerbate neuronal injury and microhaemorrhages whilst promoting the expansion of infarcts, leading to more significant disability. While rapid vessel re-cannulation to restore cerebral reperfusion is key to limiting the cytotoxic effects of energy and oxygen deprivation, it is notable that BBB leakage is prominent during reperfusion. Therapeutically, this calls for targeting endothelial pathways to promote tight junction stabilisation, and suppress transcytosis and inflammation as a means of preserving vascular barrier function, particularly in the setting of reperfusion therapies.

Collectively, these insights reinforce the pivotal role of BBB dysfunction in the pathogenesis and clinical outcomes of stroke. Nevertheless, the underlying cellular and molecular mechanisms and functional consequences remain incompletely defined, and BBB-targeted interventions are still under development. This review begins by outlining the structure and function of the BBB, including its functional integration within a neurovascular unit (NVU) and the balance between selective transport and barrier integrity. We then focus on: (1) current evidence of cerebral oedema in ischemic stroke, including the temporal evolution of cytotoxic and vasogenic oedema, (2) the emerging associated molecular signatures of BBB dysfunction (including both paracellular and transcellular pathways); and (3) the rationale for targeting the BBB to reduce oedema, inflammation, and long-term disability.

While many excellent recent reviews have explored the BBB in the context of other cerebrovascular, neurodegenerative and inflammatory diseases [2, 7, 17]the present review emphasises the stroke-specific context of BBB dysfunction and its translational relevance, with the aim to complement extensive work that focuses on other therapeutically important aspects of stroke, e.g. neuro-glial dysfunction and neuroinflammatory responses [10].

## The neurovascular unit defines functionality of the vascular BBB

The BBB operates at the level of the cerebral vasculature as well as the choroid plexus epithelium. The choroidal BBB plays important roles in the pathogenesis of many of the aforementioned neurological diseases including AD and MS. However, it only plays a subordinate role in the pathogenesis of cerebrovascular conditions such as ischemic stroke. Thus, we focus here on the vascular BBB and refer the reader to other work with focus on the choroidal BBB [18].

**Fig. 2:**
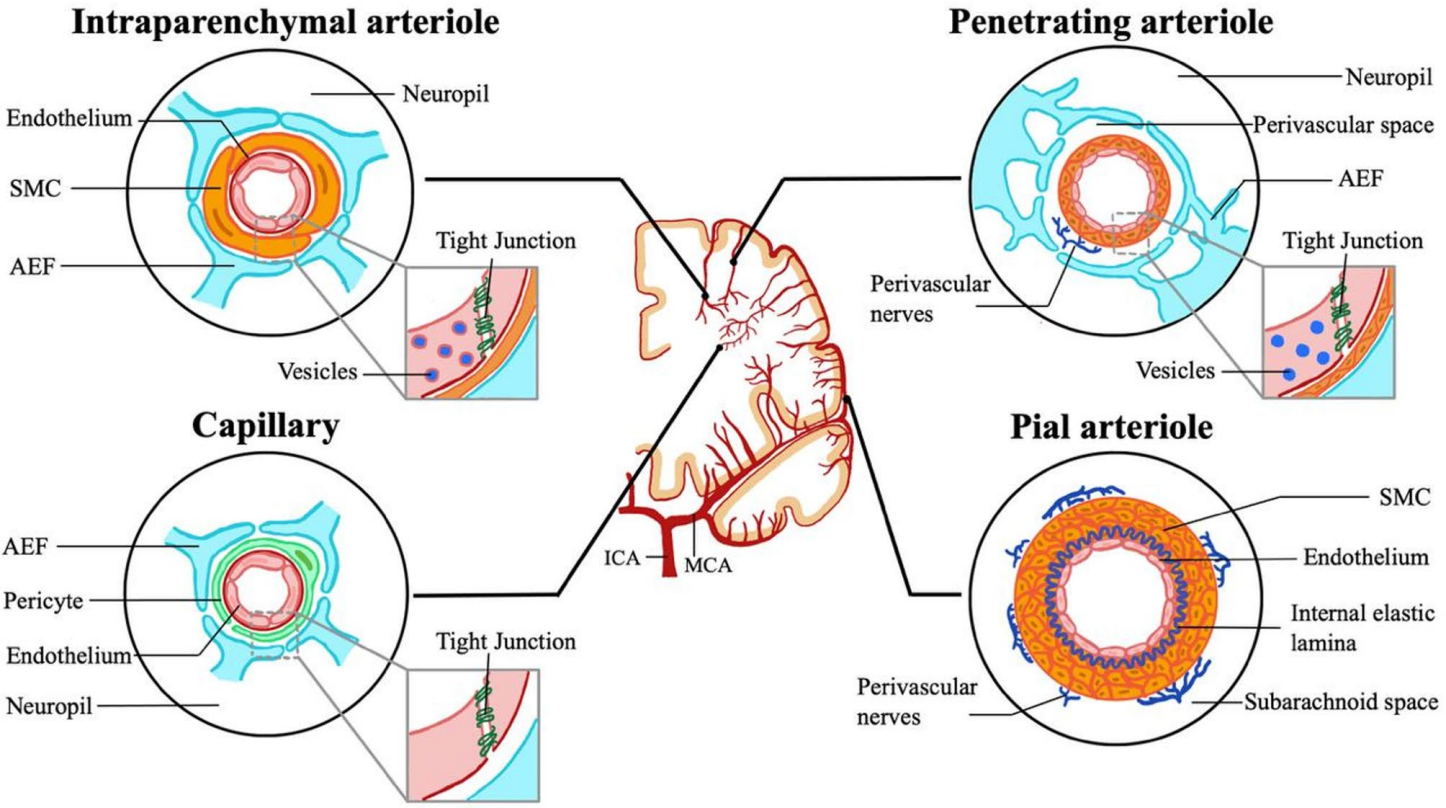
Heterogeneity of the NVU within the cerebrovascular tree. Pial arterioles on the cortical surface are innervated by peripheral nerves and have perivascular smooth muscle cells (SMCs) contributing to their role in regulation of cerebral blood flow. Penetrating arterioles dive into the cortex of the brain and are lined by a diverse perivascular space that is structurally and functionally defined by astrocyte end feet (AEF). Further into the cortex, vessels become smaller and gain BBB properties. In capillaries, SMCs are replaced by pericytes, and the presence of tight junctions and absence of pinocytosis contributes to the exquisite barrier properties seen at the BBB.

Barrier characteristics of the cerebral vasculature are not uniform and vary depending on the positioning within the vascular tree. As vessels dive into the cortex, they progressively acquire more BBB properties, with the most exquisite barrier observed in blood vessels forming the cerebral microvasculature (Fig. 2) [19]. In circumventricular organs, the microvasculature is devoid of barrier properties to enable the brain to sense changes in circulating factors and facilitate communication across the blood, cerebrospinal fluid and the brain [20]. The functional and structural diversity of ECs along the cerebrovascular tree is regulated by their abluminal interactions with a large variety of perivascular and parenchymal cells, with the interacting cytological entity now widely referred to as NVU [19, 21]. Within it a diverse range of cells and extracellular components interact to fine-tune BBB transport properties as well as other important functionalities such as neurovascular coupling. Accordingly, the neurovasculome has been identified by the American Heart and Stroke Associations as the next frontier in our understanding and the development of effective treatment of neurological diseases as wide ranging as stroke and AD [21]. The microvascular NVU comprises ECs, mural cells and glial cells. In a wider sense, neurons and immune cells also influence BBB performance and thus should also be considered part of a functional NVU.

### Endothelial cells

Lining the brain microvasculature are ECs that are phenotypically similar but also very distinct from those found in the periphery. As in the periphery, a luminal glycocalyx acts as non-selective filtration barrier and contributes to the BBB. However, unique characteristics in BBB ECs include expression of tight junction (TJ) networks connecting with adjacent ECs [22], next-to-no pinocytic activity due to the lack of caveolae at the luminal surface, and a nearly complete absence of fenestrae [23–25]. The continuous complex of virtually impermeable TJs creates a high resistance paracellular barrier preventing unregulated movement of large, hydrophilic molecules between the blood and the brain [22, 25]. The low rate of pinocytotic activity and absence of fenestrae adds a transcellular barrier. Whilst many key features of ECs are genetically predetermined (‘nature’), the exquisite barrier properties of BBB ECs are acquired during development and maintained through their NVU interactions (‘nurture’) [26]. Thus, ECs of arterioles interact with smooth muscle cells (SMCs) and display much higher pinocytic activity than those of capillaries, which are lined by pericytes (PCs) (Fig. 2) [27].

### Mural cells

Both vascular SMCs and PCs are closely associated with ECs through direct abluminal interactions and a shared vascular basement membrane. In vivo studies have shown that CNS PCs play a direct role in the regulation of critical neurovascular functions as well as integrating the functions of ECs and astrocytes at the NVU [25, 28]. These include regulation of vascular remodelling, angiogenesis, and importantly, the formation, maintenance and regulation of the BBB [28]. Certainly, dysfunctional PC endothelial interactions and PC loss has been associated with loss of BBB [28] and implicated in multiple disease states such as AD, PD and MS [5, 7, 29–31].

### Astrocytes

Glial-vascular interactions are key to the function of the NVU [32]. Indeed, CNS blood vessels are encased by astrocyte end feet covering approximately 80% of the vascular perimeter. These astrocytes are integral to the NVU and can, either directly or via the basement membrane, regulate neuronal function and coordinate signals from neurons, the BBB, and their surrounding microenvironment [25]. Additionally, they have also been shown to play a key role in influencing BBB transport properties both through their contribution to basement membrane formation, regulation of PC differentiation, and through TJ modulation via their control of expression of barrier proteins such as CLDN5 and OCLN.

### Other glial cells

Microglial and oligodendrocytes are other common glial cells present within the NVU. Microglia, the primary immune cells in the brain, regulate neuronal function and cerebral blood flow under physiological and pathological conditions [33]. Indeed, activated microglia have been shown to play a crucial role in neuroinflammation via the upregulation of various inflammation factors, and microglial phagocytosis [34]. For instance, oligodendrocyte precursor cells enhance BBB integrity and upregulation of TJ proteins in ECs through transforming growth factor-beta signalling [35].

### Neurons

Neurons in the CNS lie close to capillaries [36] and thus are also well placed to play a critical role in the regulation of microvascular permeability and cerebral blood flow. They actively regulate the permeability of the BBB via astrocyte interactions and modulation of transporters located on vascular ECs. Furthermore, they have been shown to directly contribute to TJ protein synthesis and localisation in BBB ECs [37].

## Transport and leakage across the BBB

### Paracellular transport and leakage

To protect the delicate neuronal microenvironment of the CNS, transport across the BBB is highly regulated and mostly restricted. Small, non-polar molecules, including oxygen and carbon dioxide, can freely diffuse along their concentration gradients across BBB ECs [25]. Many small (under 400 to 600 Da), diffusible, lipid-soluble molecules are often substrates of ATP-binding cassette transporter family proteins, such as P-glycoprotein, and eliminated from the NVU to protect the brain from unwanted intoxication [38]– a feature of the BBB intensely investigated in drug delivery research [39]. Any other transport of larger nutrients, metabolites and CNS regulators in and out of the brain follows highly sophisticated, molecule-specific routes that are operated either by transmembrane channels, such as those for glucose (namely glucose transporter-1), or by receptor-mediated transcytosis. Additional permeation occurs via less specific transcellular and paracellular pathways. The former describes transport directly through ECs whilst the latter pertains to transport between ECs of the BBB. Pathological vascular leakage in the CNS is usually the result of dysregulation of either of these two pathways.

Non-specific, basal permeability is likely to occur mainly via endothelial paracellular junctions since the healthy BBB lacks fluid-phase transcytosis [25]. How such molecules are transported, or diffuse, across the paracellular space at the healthy BBB is largely unknown. Most of what we know is largely inferred from the biology of the junction complexes that connect and seal membranes of neighbouring ECs and are composed of three molecularly and functionally distinct entities; adherens junctions (AJs), TJs and gap junctions. The latter are not directly involved in paracellular transport (albeit in its regulation [40]) and thus not discussed further in this review. Importantly, in ECs there is extensive crosstalk between these junctional components in the regulation of barrier integrity. Much of the scientific knowledge on junctional complexes, particularly TJs, has been derived from studies of epithelial cells and assumed to hold true for ECs [41]. However, an ever-increasing body of evidence suggests that whilst similar classes of proteins exhibit similar functions in epithelial and ECs, there are major important differences. One such difference arises from the localisation of TJ and AJ proteins which are visibly stratified in the basolateral membrane of epithelial cells but intermingled in the thin lateral areas of ECs, indicating intimate interaction to provide paracellular functionality [22].

#### Adherens junctions

It is generally accepted that paracellular junctions develop via the formation of AJs before subsequent recruitment of TJ proteins for further fortification. No current research suggests that AJs are structurally or functionally different in ECs of the periphery compared to those of the BBB. Their role in regulating leakage, however, appears to be much more prominent in the peripheries as opposed to at the BBB [42]. As such, AJs can be regarded as providing a dynamic platform for EC-EC interaction and paracellular occlusion. Central to AJ formation are homotypic interactions of transmembrane proteins of the cadherin family, specifically vascular-endothelial cadherin (CDH5). CDH5 interacts via its cytoplasmic tail with catenin adaptors to regulate the intracellular cortical (mainly actin) cytoskeleton, as well as integrate cytoplasmic and nuclear signals [43]. For instance, CDH5 expression and clustering directly promotes CLDN5 expression in ECs through a pathway involving β -catenin, the protein kinase Akt and the de-repression of the CLDN5 promoter by FoxO1 [44]. This provides direct evidence of how continuous crosstalk between AJs and TJs ensures adequate organisation and preservation of the paracellular junctions. How AJs contribute mechanistically to *basal* permeability has not been established. CDH5’s central role in vascular function was recognised in studies of mice, in which its genetic lack led to embryonical lethality at E9.5 (when the primitive network progress to a hierarchically branched, highly organized vascular network) due to severe defects in vascular remodelling [45]. Similarly to the conditional deletion of CDH5, the injection of antibodies neutralising its adhesive function induces permeability to Evans Blue in the heart and the lung [46], although crucially not in the brain [46, 47]. Inflammatory conditions and permeability-inducing factors (PIFs), such as vascular endothelial growth factor (VEGF), induce rapid phosphorylation of CDH5 and catenins, which are functionally linked to its internalisation, subsequent actomyosin contractility and enhanced permeability to both solutes and immune cells [43, 46]. This indicates that CDH5/AJs play a key role as endothelial gatekeepers, which is, however, complemented and likely superseded by a dense TJ network at the BBB.

#### Tight junctions

If AJs form the ‘glue’ holding ECs together, TJs further seal the paracellular space with a meshwork of proteins that, at the BBB, is impermeable to most but very small molecules, thus completely eliminating the free passage of lipids and proteins. TJs not only provide this elaborate ‘gate’ function but also act as ‘fence’, prohibiting the free membrane diffusion of proteins and lipids (in the extracellular sheet of the plasma membrane), which is key to the establishment of distinct apical and basal domains [48]. Ensuing membrane polarisation has mainly been studied in epithelial cells, but is likely to be emulated in ECs, where apico-basal polarisation is also a key functional feature [49, 50]. TJs are formed by a variety of transmembrane protein claudin family members, OCLN, and junctional adhesion molecules found along continuous TJ strands at endothelial cell-cell contacts, where they are also discernible as electron dense puncta in electron microscopy sections. At tricellular cell-cell contacts, additional TJ proteins make key contribution to BBB function in line with more complex architectural requirements [51]. Similarly to AJs, TJs interact with the intracellular (mostly) actin cytoskeleton and do so via adaptor proteins such as zonula occludens proteins [22, 52]. Claudins are the main constituents of TJs and the strand-forming units in TJs. There are more than 20 claudin genes, and the encoded proteins can be separated into those that seal the paracellular space, whilst others form substrate-specific pores [53]. CLDN5 is most highly expressed in cerebral ECs [54]. It is a barrier-forming claudin, which size-selectively seals the BBB for molecules > 800 Da [55]. There is continued debate about the expression of other claudins at the vascular BBB [22]. In addition to CLDN5, other claudin transcripts (CLDN1, 11, 12 and 25) have been detected at significant levels in brain capillary fractions isolated from human and mice [56]. When analysed by sc-RNAseq both CLDN11 [58] and CLDN25 [57] are detected at significant levels in ECs in addition to CLDN5. With such a restricted number of claudins found at the vascular BBB, the molecular mechanism of selective passage of ions or water through claudin pores is still unclear [53]. OCLN appears to co-polymerise with claudin strands. Its knock-out in mice produces a complex phenotype with multiorgan defects, but crucially no increase in BBB leakage [59]pointing to an accessory role in vascular permeability. How TJs regulate basal permeability and are altered during leakage is unclear, but TJ remodelling is clearly involved in the latter. A marked reduction of transmembrane TJ proteins is generally observed during paracellular leakage and thus the loss of CLDN5 and OCLN is often used as biomarker for a dysfunctional BBB [54]. Reversible phosphorylation and ubiquitination within the cytoplasmic moiety of OCLN in response to PIFs, such as VEGF, triggers its removal from the plasma membrane and thus is likely to induce major remodelling within TJ strands [60, 61]. Furthermore, increasing evidence points to endothelial transcytosis playing a key role for solute and cell permeability at the diseased BBB.

### Transcellular transport and leakage

In the presence of virtually impermeable paracellular junctions, transendothelial BBB transport activity takes centre stage to meet the high metabolic demands of the CNS, in addition to removing waste products. Generally, transcytosis can occur via phagocytosis, pinocytosis and receptor-mediated endocytosis [62]. The physiological BBB utilises mainly the latter in the form of adsorptive- as well as receptor-mediated transcytosis [38]. Adsorptive-mediated transcytosis applies largely to cationic proteins and involves yet to be characterised carriers. Receptor-mediated transcytosis operates via classical clathrin-coated vesicles, with those carrying transferrin receptors arguably the most studied at the BBB due to their usefulness for vectorised drug-transport to the CNS [39]. In general, substrate-specific transendothelial transport supports brain homeostasis and does not contribute overtly to leakage during pathology. Instead, it is non-specific blood-to-brain pinocytosis, normally suppressed at the healthy BBB, that often appears during pathology, thus contributing significantly to pathological leakage.

### Molecular and cellular features important for transcellular regulation

In the late 1950s, electron microscopy studies described that the plasma membrane of ECs, and many other cells, was frequently caved inwards to form bulb-shaped invaginations termed ‘caveolae (intracellulares)’ [63]. Subsequent studies using electron-dense tracers established that caveolae can pinch off into the cytoplasm and form uncoated, circular vesicles with a diameter of around 70 nm. This led many researchers in the 1970s to posit caveolae as the primary vehicles of both endo- and transcytotic pinocytosis. In the meantime, caveolae have been structurally and functionally well characterised [64]. Caveolins are a family of integral protein that are essential for the induction of caveolar pits. Genetic studies using loss-of-function mutants of CAV1, a key protein hub on caveolae, proved particularly insightful in pointing to a plethora of functions, including many not linked to pinocytosis. Cavin family proteins are also important, especially for inducing the characteristic curvature, tubulation and budding during vesicular traffic. To date, caveolae have been found to assume key functions in the plasma membrane [64]. These include, mechanosensing and the provision of a membrane reservoir, lipid and cholesterol regulation and trafficking, as well as regulating signal transduction by acting as scaffolds for many signal transduction enzymes. In ECs, the dependency of endothelial nitric oxide synthase (NOS3) activity on caveolar association is particularly well studied [65]. Caveolar-dependent endocytosis has been shown for albumin and integrins but a general function in pinocytosis linked to vascular leakage has long been missing [64]. Basal vascular permeability in CAV1 knockout animals (which lack caveolae) is increased, suggesting that caveolae are not responsible [66]. However, PIF-induced leakage is reduced in the brain and the periphery in animals lacking caveolae, demonstrating a role in regulating agonist-induced leakage of blood vessels [67]with some recent studies also supporting a direct role in the transcytosis of blood-borne solutes by BBB caveolae [16, 24]. Nevertheless, studies of caveolar transcytosis are complicated by the many other functions that caveolae assume. For instance, NOS3 (and thus caveolae) is involved in the regulation of AJs and TJs and the opening of the paracellular space [68]. Furthermore, whilst CAV1 is a good biomarker for caveolae, it also fulfils many non-caveolar functions [69]. As such, the experimental evidence of CAV1 in a pathophysiological situation is not sufficient to demonstrate transcytotic leakage in ECs. Nevertheless, the discovery of a key regulator of caveolae in ECs has accelerated our understanding of the cell biology of caveolae at the BBB over the last decade.

#### Suppression of caveolae at the BBB

In contrast to peripheral endothelium, the cerebral microvascular ECs display very low numbers of caveolae. In fact, the absence of caveolae correlates with, and is functionally dependent on, the presence of pericytic mural cells [28]which induce the expression of the BBB-specific MFSD2A in the abutting ECs via paracrine WNT signalling [70] (Fig. 2). MFSD2A is a lipid symporter that flips lysophosphatidylcholines from the outer to the inner leaf of the luminal plasma membrane of BBB ECs [71]. By doing so, it is critically responsible for the uptake of docosahexaenoic acid into the brain. Importantly, the ensuing accumulation of specific lysolipids in the inner leaf of the membrane appears to be sufficient to displace cholesterol and CAV1 thereby suppressing the formation of caveolae and limiting fluid phase transcytosis across the BBB and into the CNS [71]. Animal studies of early development have shown that formation of the functional BBB and blood-retinal barrier occurs *after* the establishment of TJs at cell-cell junctions *during* the suppression of endothelial transcytosis in response to MFSD2A expression [72, 73]. This correlates with observations in human foetuses where the BBB appears to be functional in the developing brain as early as the 12th week of gestation with a near complete absence of albumin transcytosis [74]. In turn, during healthy ageing the BBB becomes less effective with increased caveolar transcytosis occurring alongside a specific loss of PCs in old mice [9]. Recognition of this inverse interdependency between caveolar transcytosis and barrier integrity across life provides insight into the pathological consequences when these processes are disturbed, including an increase in BBB endothelial transcytosis seen during brain trauma [16, 75, 76].

#### Caveolae as mediators of neurovascular coupling

Large numbers of caveolae are consistently found on healthy brain arterioles (Fig. 2), where they are involved in the constriction and dilation of the vessels in response to neuronal signals; a key cerebral process known as neurovascular coupling [77]. Whilst CAV1 is required for this neurovascular coupling, NOS3 is not. Low expression of the caveolar suppressor MFSD2A is observed in arteriolar ECs as would be expected given that they are lined by SMCs and not PCs (Fig. 2). Indeed, ectopic expression of MFSD2A in cerebral arterioles reduces caveolae and neurovascular coupling, demonstrating that the functional interaction between MFSD2A and caveolae is maintained outside BBB capillaries. Additionally, more recent evidence has highlighted that arteriolar caveolae are indeed transport competent and actively involved in transcytosing molecules from the blood across the endothelium [78]. Interestingly, this permeability is bi-directional, with reverse transcytosis likely alleviating the effects of non-selective leakage caused by caveolar traffic involved in neurovascular coupling [78].

#### Caveolae support transmigration of immune cells

Infiltration of cells, e.g. immune cells, occurs at low levels into the healthy brain [79]potentially via paracellular junctions. However, most of the conclusive EM data on transmigrating immune cells has been acquired in inflammatory models, where transmigration is much more frequently observed. In these models, immune cells transmigrate across the endothelium of the BBB (or the functionally similar blood-retinal barrier) at sites close to, but not through, the TJs (which remain intact) [80, 81]. In experimental EAE, used to model hallmark inflammatory events in MS [81, 82]transmigration of immune cells occurs in post capillary venules that would normally be devoid of caveolae. Elegant use of two-photon microscopy in *Tg eGFP-Claudin5*^*+/−*^ and CAV-1 knockout mice demonstrates that CAV1-independent TJ remodelling, which is associated with paracellular leakage, precedes the neurological manifestations of EAE. By contrast, at peak EAE, BBB leakage and Th1 (but not Th17) cell migration is CAV1-dependent, indicating that sequential breakdown of the paracellular and then transcellular BBB occurs during this model autoimmune neuroinflammatory disease [82].

### Functional independence of para- and transcellular leakage at the BBB

Leakage through junctions is fundamentally different from that via transcytotic vesicles in two key areas: (1) opening of junctions creates a direct fluid phase passage between the blood and the parenchyma, whilst transcytosis does not; (2) leakage through junction is size-selective, whilst BBB caveolae transport molecules of varying size at the same rate [83]. In addition, whilst both pathways may operate simultaneously, there is also clear indication that they do so sequentially at different points during neurological and cerebrovascular disease. For instance, VEGF stimulation induces immediate junction opening at the BBB [84] but also induces caveolae in response to chronic treatment [85]. How these processes temporally overlap and functionally interact is generally not known. Thus, identifying mechanisms, biomarker and PIFs specific for each of the two pathways will be a crucial advance in the quest for bespoke anti-leakage therapies.

Lysophosphatidic acid (LPA) is a bioactive phospholipid produced by activated platelets which is present in most neuronal cell types i.e., neurons, Schwann cells, adipocytes, and fibroblasts [86]. Elevated levels of LPA are associated with several pathological states including neurological disorders. It induces very rapid (likely paracellular) permeability of the BBB [86–88]. LPA treatment of cultured mouse and human brain microvascular ECs leads to phosphorylation of TJ proteins (including CLDN5 and OCLN) as well as activation of the Rho-ROCK pathway leading to alterations in the cellular cytoskeleton, indicative of paracellular leakage [89, 90]. Indeed, transendothelial electrical resistance measurements show that LPA produces transient opening of the BBB in vitro, while the transcytosis of tracers is unaffected [83]further indicating that LPA is a paracellular-selective PIF. Methamphetamine induces BBB opening in vitro and in vivo. Whilst high concentrations are associated with TJ remodelling (presumably driven by an inflammatory response) [91]lower-dose methamphetamine has been found to upregulate caveolae-mediated transport at the BBB whilst leaving junctions intact and closed [24, 83, 92]. Collectively, these findings suggest that PIFs exist that are specific for each pathway and can be used as paradigms to uncover the fundamental machineries that independently drive each pathway.

## Cerebral oedema during stroke

Ischemic stroke, accounting for approximately 85% of all strokes [93]is characterised by the sudden loss of neurological function due to an obstruction of cerebral blood flow, typically caused by a thrombus or embolus. Despite its complex pathophysiology, both clinical and experimental findings consistently highlight sustained oedema as a hallmark feature, contributing to dysfunction and destruction of neuronal tissue, neuroinflammation and adverse prognoses and clinical outcomes [94, 95].

**Fig. 3:**
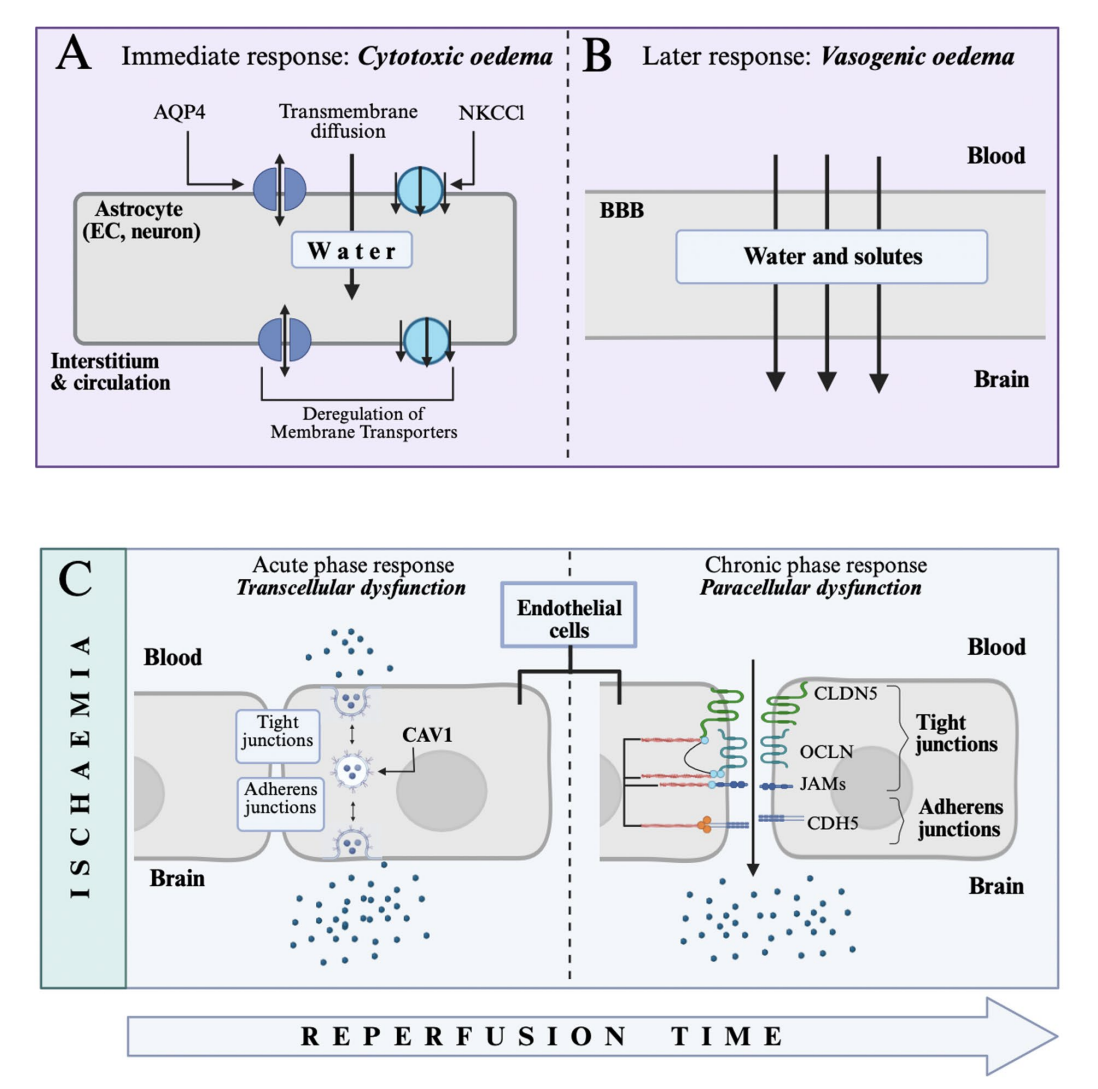
Physiological and molecular mechanisms underlying cerebral oedema during ischaemic stroke. (**A**) Cytotoxic oedema occurs in response to energy deprivation and leads to water ingress mainly in astrocytes but also neurons and ECs. It is predominant in the acute response. Important contributors (non-exhaustive) are AQP4 water channels and NKCCl cotransporters. (**B**) Vasogenic oedema is observed later and involves BBB breakdown and water and plasma solute efflux from the circulation into the parenchyma. (**C**) Proposed endothelial cell biology of BBB breakdown as observed in experimental MCAO. Biphasic leakage during reperfusion involves transcellular dysfunction (i.e. increased transcytosis) succeeded by paracellular dysfunction (i.e. opening of TJs and AJs). Made with BioRender

Clinically, ischemic stroke manifests with focal neurological deficits, including weakness, speech impairment, or visual disturbances, depending on the affected region of the brain [96]. Imaging studies reveal a characteristic progression of pathology; early cytotoxic oedema, detectable within minutes via diffusion-weighted MRI, reflects cellular swelling due to water ingress in neuro-glial cells that respond to energy failure [97]. As the stroke evolves, particularly with reperfusion, BBB compromise leads to vasogenic oedema which is characterised by water and solute efflux from the circulation into the extracellular space (Fig. 3A, B). This phased evolution and shift from a reduction to an increase of parenchymal extracellular fluid underpins the worsening of oedema burden, which, in severe cases, contributes to malignant middle cerebral artery syndrome.

### Cytotoxic versus vasogenic oedema

Cerebral oedema in stroke is a dynamic and multifactorial process. Whilst cytotoxic or vasogenic oedema can be distinguished accurately by diffusion-weighted MRI [98]their precise timing and progression during ischemic stroke remains unclear due to a shortage of systematic imaging studies in acutely ill patients. Clearly, they often overlap, evolve over time and are functionally interconnected.

Cytotoxic oedema emerges rapidly after ischaemic onset, within the first 6 h, and reflects the metabolic phase, when neuro-glial cells respond to energetic deprivation resulting in ATP depletion, failure of Na⁺/K⁺-ATPase activity, and dysregulation of ion haemostasis [12]. Water follows osmotic gradients to induce cellular swelling of astrocytes, but also other glial cells, as well as neurons and ECs (Fig. 3A). Important contributors include Na-K-2Cl co-transporter-1 (NKCC1) and aquaporin-4 (AQP4) water channels, particularly in astrocyte end feet. Notably, while cytotoxic oedema is not a result of failure of the BBB, cell swelling, especially of astrocytes and subsequent detachment of their end feet may prime TJ destabilisation in the BBB and subsequent leakage [3, 14]illustrating the functional interconnection of different forms of cerebral oedema.

In contrast, vasogenic oedema reflects direct BBB breakdown and coincides with inflammatory and vascular responses (Fig. 3B, C). It features prominently during reperfusion, is generally observed 24- and 72-hours post-stroke and involves both paracellular and transcellular leakage. The former is driven by TJ degradation (e.g. of CLDN5, OCLN, ZO-1) via matrix metalloproteinases (e.g. MMP-9) and inflammatory mediators [10, 13, 99]whereas the latter is marked by increased CAV1 activity and suppressed MFSD2A expression [15, 100, 101]. These changes permit plasma proteins and fluid to extravasate into the interstitial space, increasing intracranial pressure and worsening tissue injury [102, 103].

Importantly, the resolution of oedema requires a number of mechanistically diverse processes. Neuro-glial integrity must be restored including the recalibration of ion gradients and the recovery of water clearance mechanisms, particularly AQP4 polarity and astrocytic function. At the same time permeability-inducing pathways must be attenuated and the BBB restored. However, while a leaky BBB contributes to oedema it is likely also important in providing an exit route for excess fluid. Therefore, it is important to understand the mechanistic and functional underpinnings of BBB breakdown during the more chronic phase of stroke.

### Experimental models of ischemic stroke and BBB dysfunction

It has been increasingly recognised that BBB permeability underlying pathological injury in ischaemic stroke is biphasic in nature, directly contributing to the phased evolution of oedema mentioned above. More recently, it has been suggested that this biphasic leakage is mediated by hierarchical activation of transcellular and paracellular pathways with initial, mild, barrier dysfunction resulting from transcellular leakage via increased caveolar transcytosis, and secondary, more severe damage, characterised by TJ protein remodelling and increased paracellular permeability [16] (Fig. 3C). Initial evidence confirming this hypothesis was derived from ultrastructural analysis of TJ integrity following MCAO in rats in the acute phase of ischaemic stroke which showed minimal disruption of TJs in areas with massive albumin leakage [104]. Further reports with transgenic mouse models of stroke with eGFP labelled ECs have similarly found that upregulation of caveolae-mediated transcytosis, followed by delayed and prolonged disassembly of TJ complexes, underlies BBB dysfunction in MCAO [16].

It is important to recognise discrepancies in results from ischaemic stroke models. Biphasic leakage is not universally observed as the sole mechanism of BBB disruption. Another study suggests that CAV1 mediated leakage (i.e. trans-endothelial vesicular transport) does not contribute to BBB disruption until up to 24 h post-ischaemia [105]. Instead, BBB disruption is driven by actin polymerisation (resulting in junctional protein redistribution) followed by MMP-9 activity degrading junctions and the extra-cellular matrix. These discrepancies may be due to differences in the genetic background of the mice as well as the mechanics and severity of ischaemic insult [16]. Indeed, MCAO models show considerable discrepancies in occlusion times across studies (varying from just 20 min to permanent occlusion) [16, 75, 105–107]and thus crucial differences in length of ischaemia, endothelial hypoxia (and potentially cytotoxic oedema). Notably, most studies enlisting shorter occlusion times tend to observe initial transcytotic leakage, whereas those with longer occlusion times are more likely to report predominant junction breakdown. In addition to these discrepancies, the question also remains how occlusion times in MCAO in rodents correlate to that in humans. Further studies are warranted in order to determine a specific MCAO protocol with an occlusion time relating to maximal leakage-induced damage whilst maintaining acceptable survival rates.

Whilst there is evidence for BBB dysfunction - biphasic or otherwise - following ischaemic stroke in animal models, current evidence in humans is more limited in nature. Evidence in patients is essential to confirm the exact mechanism underlying leakage in ischaemic stroke for specific targeting in leakage prevention. The most important causative factor in the failure of translation of pre-clinical to clinical studies lies within the ethical and logistical implications and considerations of human experimentation that are almost impossible to navigate. These involve either inducing ischaemic stroke in human patients or immediately imaging patients following ischaemic stroke in lieu of prioritising their treatment. In one study, sequential MRIs were taken in human acute ischaemic stroke in order to document early BBB changes [108]. Additional studies with similar focuses are required in order to conclusively elucidate the temporal mechanisms of BBB leakage following ischaemic stroke [109].

## Therapeutic approaches to treating stroke

**Fig. 4:**
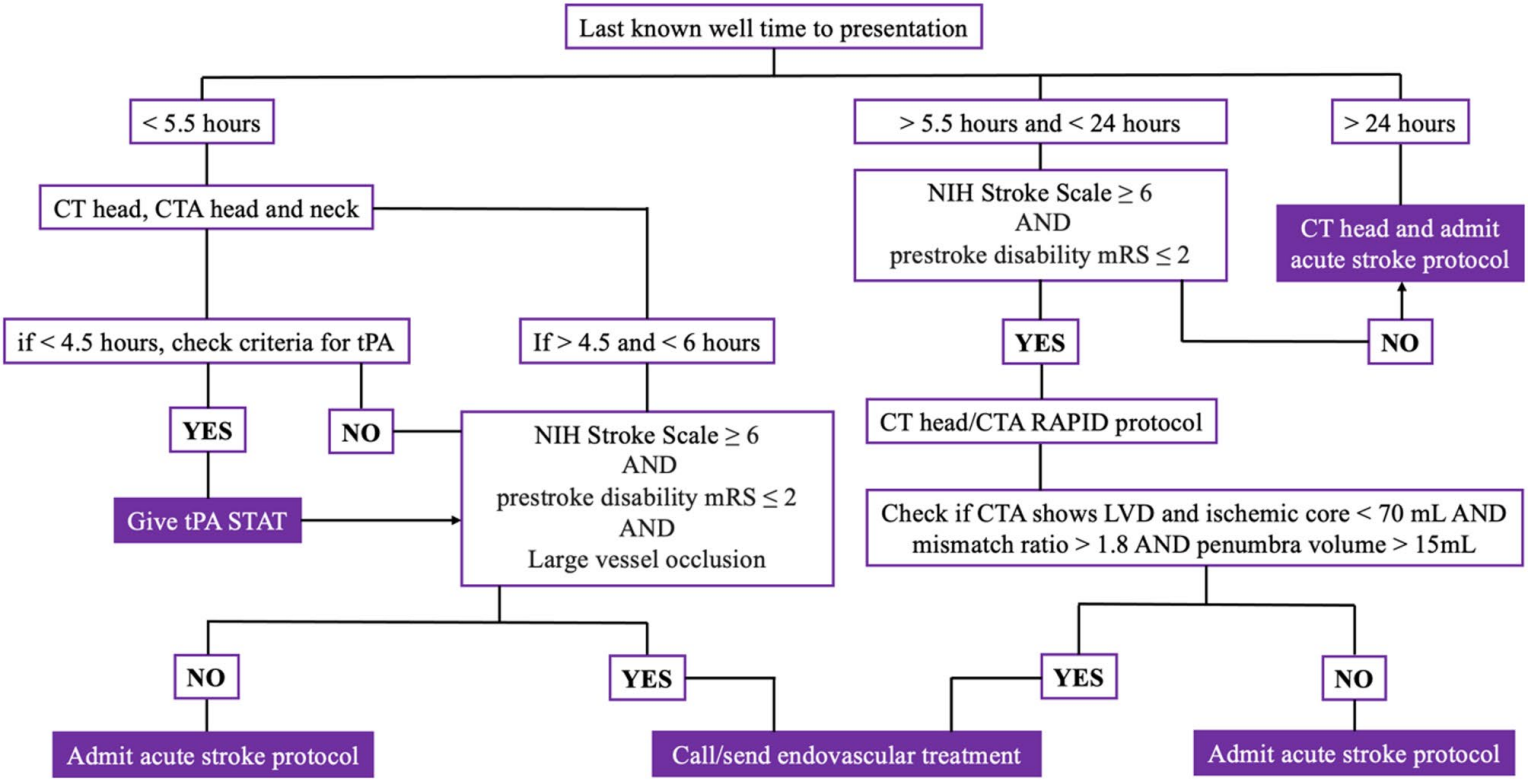
An illustrative algorithm for acute ischaemic stroke management. Usually this is institution specific, therefore should only be used as a sample guideline. CT = computed tomography; CTA = computed tomography angiography; LVO = large vessel occlusion; mRS = modified Rankin Scale; NIH = National Institutes of Health; tPA = alteplase. Adapted from [113].

Current guidelines for acute management of stroke and transient ischaemic attack [110] are focused on the imminent restoration of cerebral perfusion (Fig. 4). This is generally achieved with an initial loading dose of aspirin 300 mg, unless contraindicated, followed by intravenous thrombolysis and/or endovascular thrombectomy if eligible, and admission to a stroke unit. Currently, the only universally approved thrombolytic agent for acute ischemic strokes is Alteplase [111, 112]which is most effective within the first 4.5 h after stroke onset. For patients suffering from large vessel occlusion, endovascular thrombectomy is recommended within 24 h from symptom onset. Once the patient is in the stroke unit, secondary preventative strategies are initiated, including antiplatelets, statins, and vascular risk factor management. Approximately 10–20% of stroke patients admitted to a stroke unit may receive thrombolysis, and about 5–10% may undergo thrombectomy, with some overlap between the two in certain cases. These numbers can vary based on regional and institutional practices.

### Key therapeutics for targeting ischemic stroke

Understanding the underlying mechanism of ischaemic stroke-related BBB dysfunction is essential for developing targeted interventions to better manage this pervasive condition.

Therapies targeting BBB dysfunction in ischaemic stroke are diverse, and focus on reducing inflammation, oxidative stress, and endothelial damage. Largely, it is thought that disruption of TJs and increased barrier permeability, characteristic of stroke, is brought about by soluble factors releasing various mediators into the hypoxic tissue. These include proinflammatory cytokines, nitric oxide, matrix metalloproteinases and VEGF [1–3, 114]. Direct targeting of some of these mediators has been the focus of numerous preclinical trials to-date. Select examples are outlined in Table 1.

**Table 1:** Non-exhaustive list of current therapeutics targeting leakage underlying ischaemic stroke

Current therapeutics targeting leakage underlying ischaemic stroke				
**Pharmacological therapies**	**Cell-based therapies**	**Targeting BBB disruption mechanisms**	**Additional**	**Emerging therapies**
Matrix metalloproteinase inhibitors *(e.g. Minocycline*,* Doxycycline)* Anti-Inflammatory Agents *(e.g. Corticosteroids*,* Tocilizumab)* Endothelial Tight Junction Stabilisers *(e.g. Statins - Simvastatin)* Oxidative Stress Modulators *(e.g. N-acetylcysteine*,* Edaravone)*	Mesenchymal Stem Cells Exosome Therapy	Inhibition of AQP4 Sphingosine-1-phosphate receptor modulators *(e.g. Fingolimod)*	Hypothermia	Wnt/β-Catenin Pathway Activation Nanomedicine

While many promising approaches are being explored, most remain in preclinical or early clinical stages, with some yielding mixed results [115]. This highlights the need for a deeper understanding of the mechanism behind BBB dysfunction and its downstream effects, crucial for developing effective targeted therapies. Current therapeutic targets being explored are expanded on further below.

### Therapeutic targeting of inducers of cerebral oedema

Despite evolving research demonstrating the grave effects of cerebral oedema and brain swelling on patients’ functional outcomes after stroke, current standard practice for stroke management does not involve targeting of the specific molecular mechanisms involved in cerebral oedema. Moreover, medical management of cerebral oedema is largely centred around differences between cytotoxic and vasogenic oedema – a distinction which is becoming increasingly artificial [12]. Research using animal models of ischemic stroke have helped to improve understanding of the complicated mechanisms underlying cerebral oedema in stroke and aided in identifying several potential therapeutic targets. Additionally, there have been reports of large-scale human trials designed to investigate these targeted interventions [116].

#### Targeting ion transporters

Bumetanide is a loop diuretic generally used for the symptomatic relief of chronic heart failure by reducing fluid retention [117]. More recently it has been postulated that due to its role as a direct antagonist of the Sodium-Potassium-Chloride co-transporter (NKCC1) it may also prove effective in reducing astrocyte swelling and hence reduce amounts of cytotoxic derived cerebral oedema. In vitro studies have not only shown that cerebral tissues express higher concentrations of NKCC1 [118], but that under hypoxic conditions there is further upregulation of these channels suggesting a direct role of NKCC1 in cerebral oedema formation. Although there are currently no reports of bumetanide use in humans to control cerebral oedema, several animal studies have shown a degree of improvement in oedema following administration of bumetanide [12, 118, 119].

Upregulation of Sulfonylurea receptor 1 following cerebral injury has been shown to induce the formation of the nonselective cation channel, SUR1-TRPM4, resulting in intracellular movement of water and subsequent oedema [12, 120, 121]. Targeting of this channel using sulfonylurea binding drugs such as glyburide has therefore been investigated in human trials and have shown short term functional improvements in patients following traumatic brain injury [122]. Other sulfonylurea binding drugs such as Glibenclamide have also been shown to be indirect inhibitors of MMP-9 thus potentially impacting on vasogenic oedema/BBB dysfunction. With increasing evidence highlighting the key role of MMP-9 in stroke, selective inhibition using Glibenclamide to reduce and manage cerebral oedema has become more readily researched [116]. It is important to note however that due to the presence of MMP’s throughout the body, targeting of such molecules could lead to severe undesired secondary effects.

#### Targeting of aquaporins

Aquaporins exist throughout the human body and facilitate the movement of water across cell membranes. AQP4 is the most abundant aquaporin within the brain and is largely localized to astrocyte end feet [12, 123, 124]. Although there has been conflicting evidence for the role of APQ4 in murine models of traumatic brain injury, it is generally agreed that deletion and/or inhibition of aquaporins reduce cytotoxic oedema [124, 125]. Whilst it is recognised that this may also worsen vasogenic oedema, AQP4, and its selective inhibitors AER-270 and AER-271, have been explored as potential therapeutic target in the management of cerebral oedema. Indeed, phase 1 of a clinical trial investigating AER-271 for use in treatment of acute ischemic stroke has now been completed, but results have not yet been published *[ClinicalTrials.gov Identifier: NCT03804476].*

#### Targeting VEGF

As mentioned in the above sections, VEGF is a PIF that plays a key role in BBB permeability and research has confirmed that there is large scale upregulation of VEGF in peri-infarct regions following onset of ischemia. The inhibition of VEGF has therefore been extensively researched as a therapeutic target for preserving BBB integrity. Indeed, inhibition of VEGF using antibodies and molecular traps [126] has been considered in attempts to reduce cerebral oedema. In mouse MCAO, cortical oedema is reduced following prophylactic intraperitoneal treatment with a VEGF trap [127]. In rat MCAO, intracerebroventricular delivery of anti-VEGF antibodies early during reperfusion reduces infarct size, BBB leakage and brain oedema thus improving neurological outcomes [128]. In contrast, intracerebroventricular application of a VEGF agonist, namely a mimetic which enhances angiogenesis but not vascular permeability, is neuroprotective in rat MCAO [129]. Thus, both attenuation and stimulation of cerebral VEGF signalling may be beneficial in a setting of ischemia-reperfusion and more detailed studies are required to understand underlying molecular mechanisms. Caution should also be exerted when considering anti-VEGF treatment in cerebrovascular disease as its neutralisation could also affect neurogenesis [130] as well as long-term vascular homeostasis [131, 132]. Additionally, since VEGF receptors mediating BBB permeability are located abluminally, systemic anti-VEGF treatment would require a severely compromised BBB to be effective [50].

### Blocking BBB leakage directly to treat cerebrovascular disease

Most of the above approaches focus on neutralising signalling factors or mediators that enhance leakage pathways; however, these often have pleiotropic effects in a wide variety of cell types within and outside the NVU. Targeting PIFs requires patho-mechanistic knowledge, which is clearly still missing for ischaemic stroke, and may also vary between patients. More recently it has been suggested that BBB leakage itself should be targeted and, given the evidence for mechanistically distinguishable phases, interventions that are specific for either the paracellular or caveolar pathways.

#### Regulation of CLDN5 expression to stabilise the BBB and prevent seizure activity

Theoretically, all components contributing towards TJs between ECs could be considered as targets to modulate TJ function, thus directly targeting junctional leakage. Importantly however, targeting specific components of TJ complexes may oftentimes cause more harm than good, where loss of adhesion molecules, for example, is associated with carcinogenesis [133].

Currently, there is a limited body of evidence supporting strategies exclusively targeting junctional transport. Within this narrow body of research, a consistent finding is the critical role of CLDN5 in maintaining TJ integrity and thus in strongly regulating paracellular permeability [55, 133]. Experimental animal models of epilepsy have described a wide range of BBB abnormalities including TJ disruption and increased immune cell infiltration, indicating a strong degree of correlation between BBB insult and breakdown with seizure activity [134, 135]. Emerging research reported seizures in animal models with brain-wide depletion of CLDN5 as well as evidence of spontaneous recurrent seizures in CLDN5 knockdown mice [136]. Conversely, restoration to physiological levels of CLDN5 was found to reduce seizure activity and neuroinflammation [136]. Data from the same study suggests that downregulation of CLDN5 promotes upregulation of cell adhesion molecules resulting in immune cell trafficking and BBB breakdown.

Transforming growth factor beta pathway inhibitors, such as RepSox, are amongst novel compounds that regulate CLDN5 expression [137]. When added to ECs differentiated from human pluripotent stem cells, RepSox improved barrier function and prevented VEGF-A induced permeability in vitro [137]. It can therefore be hypothesised that stabilisation of the BBB via upregulation of CLDN5 using individual small molecules, such as RepSox, may be an effective anti-leakage therapeutic intervention.

Taken together, the current evidence suggests that selective targeting and regulation of junctional proteins, in particular CLDN5, may represent a promising therapeutic approach for preserving BBB integrity in diseases of the CNS.

#### Regulation of CAV1 expression to modulate the BBB

Whilst targeting paracellular pathways across the BBB has mainly focused on seeking to modulate CLDN5, the focus for transcellular pathways has largely been CAV1. Direct targeting of CAV1 has been explored as a potential modality for leakage inhibition. Most studies have found that BBB disruptions, such as those seen in focal cerebral ischaemia and reperfusion injury, are associated with a down-regulation of CAV1 [138,139]. Mechanistically, there is evidence that reduced CAV1 is functionally connected to MMP up- and TJ down-regulation [139]leading to increased (paracellular) permeability in ischaemic areas. This is in line with established signalling activity of CAV1 and caveolae [140]. Indeed, genetic knockout of CAV1 exacerbates TJ downregulation, BBB dysfunction and neuronal injury [100, 106]. Conversely, overexpression of CAV1 in genetic mouse models, in the context of MCAO, reduces vasogenic oedema in the acute phase of ischaemic stroke [141]. Collectively, none of the studies focusing on CAV1 expression have uncovered the functional role of transcytosis in the pathogenesis of ischemia-reperfusion. Additionally, the dual role of CAV1 for transcytotic vesicles and in the regulation of TJ levels [140] make it an unspecific target for transcytosis at the BBB. Whilst Knowland et al. reported a reduction of transcytosis in CAV1 knockout mice, they were unable to dissociate this from the concomitant increase in paracellular BBB permeability [16]. Notably, rates of caveolar transport can be modulated in isolation by targeting MFSD2A levels in models of sub-arachnoid haemorrhage [142] or MCAO, where this influences infarct size [143]. However, the pathological consequences of only a lack of transcytosis need additional, in-depth investigation. Permanent, whole animal knockouts are unlikely to uncover critical roles of transcytosis during ischaemia-reperfusion that are also endothelial specific and predicted to be transient [140]. Therapeutic targeting of transcytosis will require the identification of molecules that are truly specific to this pathway. This could then lead to the development of much needed robust and specific anti-leakage interventions for the treatment of ischaemic stroke and other cerebrovascular diseases.

## Concluding remarks and perspectives

BBB dysfunction and leakage contributes significantly to the morbidity and mortality of cerebrovascular disease including ischaemic stroke, and understanding the underlying mechanism and functional consequences will undoubtedly further the therapeutic options available to patients. Given the complexity and diversity of the BBB breakdown that has been observed in animal models and the paucity and complications of studying cerebral oedema in acutely admitted patients, research and development towards meaningful anti-leakage interventions may benefit from a focus on some clearly deliverable objectives.

First, modelling of cerebral ischaemia may benefit from technical consolidation that focuses on how closely they are adapted to the human presentation. Given that BBB dysfunction developed with different mechanics and time courses across various studies, we are still seeking answers to how relevant reported features are to human disease.

Furthermore, identification of specific biomarkers for the different types and phases of BBB dysfunction should facilitate research on BBB dysfunction in general and help with decisions around devising and assessing meaningful models. Such biomarkers identification should also consider their translatability to acutely ill patients, as they might ultimately be the sole meaningful link between animal and human conditions.

Lastly, the pre-clinical development of BBB leakage intervention should focus on mechanistic specificity and functional outcomes: Clearly, BBB leakage is not uniform during cerebral ischaemia-reperfusion, with at least two mechanistically very different modes of leakage likely occurring at different times during pathogenesis. Wholesale blockade of leakage or even sealing the BBB may not prove beneficial to neurological outcomes as surely some permeability is required to clear insults in the form of inflammatory factors, blood constituents normally alien to the brain or excessive water accumulation. Future research should therefore adopt a more nuanced strategy, incorporating a broad range of neurological outcomes, including motor, cognitive, imaging, and oedema-related measures, rather than relying solely on infarct volume.

Taken together, recent progress in understanding molecular aspects of BBB and its regulation by the NVU on single-cell level predicates rapid advances towards more effective treatments for ischaemic stroke.

## Data Availability

No datasets were generated or analysed during the current study.

## Abbreviations

AD: Alzheimer’s disease
AJ: Adherens junction
AQP4: Aquaporin-4
BBB: Blood-brain barrier
CDH5: Vascular-endothelial cadherin
CLDN5: Claudin-5
CNS: Central nervous system
EAE: Autoimmune encephalomyelitis
EC: Endothelial cell
LPA: Lysophosphatidic acid
MCA: Middle cerebral artery
MCAO: Middle cerebral artery occlusion
MS: Multiple sclerosis
NOS3: Endothelial nitric oxide synthase
NKCC1: Sodium-potassium-chloride co-transporter
NVU: Neurovascular unit
OCLN: Occludin
PC: Pericyte
PD: Parkinson’s disease
PIF: Permeability-inducing factor
SMC: Smooth muscle cell
TJ: Tight junctions
VEGF: Vascular endothelial growth factor
